# Epidemiological trends, antimicrobial resistance, and genetic mutations of *Mycoplasma pneumoniae* in Wuhan, China: a post-COVID-19 perspective

**DOI:** 10.1515/med-2026-1400

**Published:** 2026-04-09

**Authors:** Shan Chen, Liang Luo, Lu Liu, Meng Wang, Qian Yin, Li Ma, Liu Yang, Jianglan Wang, Zhen Hu

**Affiliations:** Department of Clinical Laboratory, Wuhan Caidian District People’s Hospital, Wuhan, People’s Republic of China

**Keywords:** *Mycoplasma pneumoniae*, antibiotic resistance, 23S rRNA gene, macrolide resistance

## Abstract

**Objectives:**

This study aimed to investigate the epidemiological trends, antimicrobial resistance patterns, and genetic mutations associated with *Mycoplasma pneumoniae* infections in Caidian District, Wuhan, Hubei Province.

**Methods:**

Totally 7,234 nasopharyngeal swabs were collected from patients with respiratory symptoms. RT-qPCR was used to detect *M. pneumoniae* DNA, and antimicrobial susceptibility testing was performed on cultured isolates. The 23S rRNA gene was sequenced to identify resistance-associated mutations at positions A2063G, A2064G, A2067G, and C2617G by Pyrosequencing.

**Results:**

The overall positivity rate for *M. pneumoniae* was 19.37 % (1,401/7,234), with significantly higher infection rates in children (32.75 %) compared to adults (9.84 %). Moreover, 20.51 % of *M. pneumoniae* isolates were susceptible to all tested antibiotics, while 24.36 % exhibited resistance to a single antibiotic class, with macrolide resistance being predominant (15.38 %). Multidrug resistance was observed in 55.13 % of isolates, primarily driven by macrolide-lincosamide co-resistance (34.62 %). The mutation rate in the 23S rRNA V domain was 94.87 %, with A2063G being predominant (65.38 %).

**Conclusions:**

This study reveals a high prevalence of macrolide resistance and multidrug resistance, primarily involving macrolide and lincosamide, in clinical isolates of *M. pneumoniae*. The high mutation rate in the 23S rRNA V domain underscores the need for continuous surveillance of resistance patterns and genetic mutations.

## Introduction

*Mycoplasma pneumoniae* (*M. pneumoniae*) is a significant etiological agent of both upper and lower respiratory tract infections across all age groups, especially in pediatric and adult populations, accounting for 10–30 % of community-acquired pneumonia (CAP) cases [1]. Patients with *M. pneumoniae* infections may be afflicted with headache, sore throat, fever, cough, and other common respiratory symptoms [2]. Although *M. pneumoniae* infection is usually considered a self-limited benign disease, without early accurate diagnosis and effective treatments, some patients can develop pneumonia with extrapulmonary manifestations involving almost every organ system [3]. The absence of a cell wall in *M. pneumoniae* renders β-lactam antibiotics ineffective [4]. The current therapeutic paradigm for *M. pneumoniae* pneumonia (MPP) in both adult and pediatric populations prioritizes macrolides as first-line antimicrobial therapy [5]. Nevertheless, this therapy has led to the emergence and global spread of macrolide-resistant *Mycoplasma pneumonia* strains [6], 7]. Tetracyclines and fluoroquinolones have emerged as effective alternative therapeutic options for drug-resistant MPP, although their use in children is limited due to safety concerns [7], 8]. Therefore, given the increasing prevalence of antimicrobial resistance and the cyclical epidemic patterns of *M. pneumoniae* infections, ongoing surveillance of resistance trends and developing novel therapeutic strategies remain critical priorities in respiratory medicine.

In recent years, the global prevalence of macrolide-resistant *M. pneumoniae* (MRMP) has risen significantly, posing substantial challenges to clinical management [9]. Epidemiological studies reveal particularly high resistance frequencies in Asia, where approximately 80–90 % of clinical isolates in China harbor macrolide resistance mutations [9], 10]. This resistance primarily arises from point mutations in the 23S rRNA, particularly A2063G and A2064G in domain V, which reduce macrolide binding affinity [11]. The geographical variation in resistance prevalence likely reflects differences in antibiotic prescribing patterns and host population characteristics. A comprehensive understanding of regional resistance profiles is critical for optimizing treatment regimens, mitigating antibiotic selection pressure, and developing targeted strategies to contain the spread of resistance.

Based on the above evidence, this study aims to investigate the prevalence, phenotypic patterns, and 23S rRNA gene mutations associated with antimicrobial resistance in *M. pneumoniae* isolates from pediatric and adult patients in Wuhan in the post-COVID-19 era. Our findings may provide an in-depth understanding of the epidemiology of *M. pneumoniae* in this region after the COVID-19 epidemic.

## Materials and methods

### Study population

A total of 7,234 patients, including 3,008 children (<18 years of age) and 4,226 adults (≥18 years of age), who had attended the outpatient and inpatient departments of Pediatrics and Respiratory and Critical Care Medicine of Wuhan Caidian District People’s Hospital between June 2023 and June 2024 and presented with respiratory tract infection symptoms, were enrolled in this study. The exclusion criteria are as follows: 1) Hospitalization for *M. pneumoniae* infections within the preceding month. 2) History of unresolved severe pneumonia or active multi-organ dysfunction. 3) Severe comorbidities (hepatic, renal, cardiovascular, or hematopoietic disorders) or ongoing immunotherapy.

### Specimen collection

Nasopharyngeal swab (NS) samples were obtained from the enrolled patients within 24 h after admission for *M. pneumoniae* screening. Concurrently, serum samples were obtained to detect procalcitonin (PCT), interleukin-6 (IL-6), and C-reactive protein (CRP) using ELISA kits (CUSABIO, Wuhan, China). Whole blood samples were also collected for complete blood count (CBC) analysis.

### *Mycoplasma pneumoniae* nucleic acid test

DNA was extracted from all NS samples using a DNA Mini-kit (Tianlong Cp., Ltd., Chongqing, China) following the manufacturer’s instructions. *M. pneumoniae* nucleic acids were detected using a PCR Fluorescence Probing kit (Shengxiang Co., Ltd., Hunan, China). The PCR amplification system consisted of 10 μL DNA template, 38 μL MP PCR reaction mix, and 2 μL Taq enzyme. Amplification was performed on a quantitative PCR instrument (ABI Prism 7,500, Applied Biosystems, Foster City, USA) with the following thermal cycling protocol: denaturation at 94 °C for 5 min, followed by 45 cycles of denaturation at 94 °C for 15 s and annealing/extension at 57 °C for 30 s, with a final step at 25 °C for 10 s.

### Bacterial culture and antimicrobial susceptibility testing

From all PCR-positive cases, 484 samples were randomly selected for *M. pneumoniae* culture using a computer-generated random number sequence in Microsoft Excel. The culture-positive isolates obtained were used for subsequent antimicrobial susceptibility testing. Before inoculation, both the culture medium and antimicrobial susceptibility testing plates (Antu Bio, Zhengzhou, China) were equilibrated to room temperature. Then, 100 µL of sterile culture medium was aliquoted into the control well. NS samples were vortexed thoroughly in the remaining culture medium, and 100 µL of the sample-containing medium was dispensed into each remaining well of the plate. The plate was gently agitated to ensure uniformity, followed by the addition of one drop of mineral oil to each well to prevent evaporation during the extended incubation period. The plate was then sealed and incubated at 37 ± 1 °C for 48 h. Results were interpreted based on color changes of the culture medium following the manufacturer’s instructions: the quality control well turning yellow indicated positivity (*M. pneumoniae* growth), while reactivity was indicated by a color change from red to yellow in test wells. Resistance was defined by color changes in both upper and lower wells, whereas an intermediate result was indicated by the upper well turning yellow and the lower well remaining red. The test evaluated susceptibility to 14 antimicrobial agents across four classes: macrolides (erythromycin estolate, erythromycin, azithromycin, josamycin, acetylspiramycin, clarithromycin, roxithromycin), tetracyclines (minocycline, doxycycline), quinolones (ciprofloxacin, moxifloxacin, levofloxacin, gatifloxacin), and lincosamides (clindamycin).

### Pyrosequencing of drug-resistant gene loci

Pyrosequencing is a DNA sequencing technique based on an enzyme-coupled chemiluminescent reaction involving four enzymes: DNA polymerase, ATP sulfurylase, luciferase, and apyrase. Specific primers targeting the 2,063–2,067 and 2,617 loci in the 23S rRNA gene of *M. pneumoniae* were designed and mixed with template DNA in a reaction solution. The process was initiated with thermal denaturation at 95 °C to separate DNA strands, followed by primer annealing at 58 °C to facilitate specific hybridization. During the sequencing phase, dNTPs were sequentially introduced to the reaction system. Each nucleotide incorporation event, catalyzed by DNA polymerase, releases pyrophosphate (PPi) molecules. The ATP sulfurylase enzyme immediately converted these PPi molecules into ATP, which subsequently served as the energy source for luciferase-mediated oxidation of luciferin to oxyluciferin, generating detectable light signals. Between nucleotide additions, apyrase efficiently degraded any unincorporated dNTPs to maintain reaction specificity. The precise timing and intensity of the emitted light signals were recorded in real time by the pyrosequencing instrument (Qiagen, Germany), allowing for the accurate determination of the DNA sequence at each target locus.

### Statistical analysis

The data in this study were analyzed using SPSS 26.0 software (IBM Corp., Armonk, NY). The data were tested for normality using the Shapiro-Wilk test. Normally distributed quantitative data were expressed as mean ± standard deviation and compared using independent samples *t*-tests. Non-normally distributed data were presented as median and interquartile range and compared using non-parametric independent samples tests. Qualitative data were analyzed using chi-square or Fisher’s exact tests to examine the relationship of resistance rates among different populations and sexes. A p-value<0.05 was considered statistically significant, except when a Bonferroni adjustment was applied.

### Ethical approval

This study was approved by the Ethics Committee of Wuhan Caidian District People’s Hospital (Approval No. LLSC2024051521). Written informed consent was obtained from patients or their parents.

## Results

### Distribution of *M. pneumoniae* infections in different months and age groups

**Figure 1:**
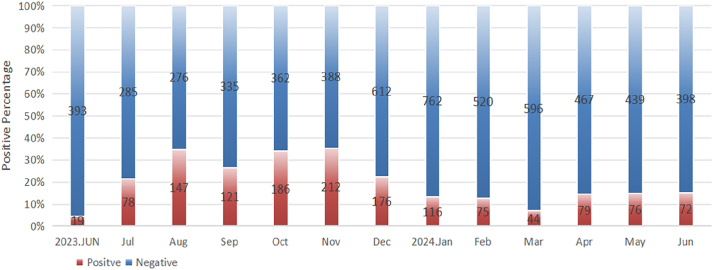
Monthly distribution of positive and negative *Mycoplasma pneumoniae* case numbers in Wuhan, China, from June 2023 to June 2024. The blue bars represent negative samples (total n=5,833), while the red bars indicate positive samples (total n=1,401). The number of positive samples and negative samples was shown by simple enumeration in each month.

**Figure 2:**
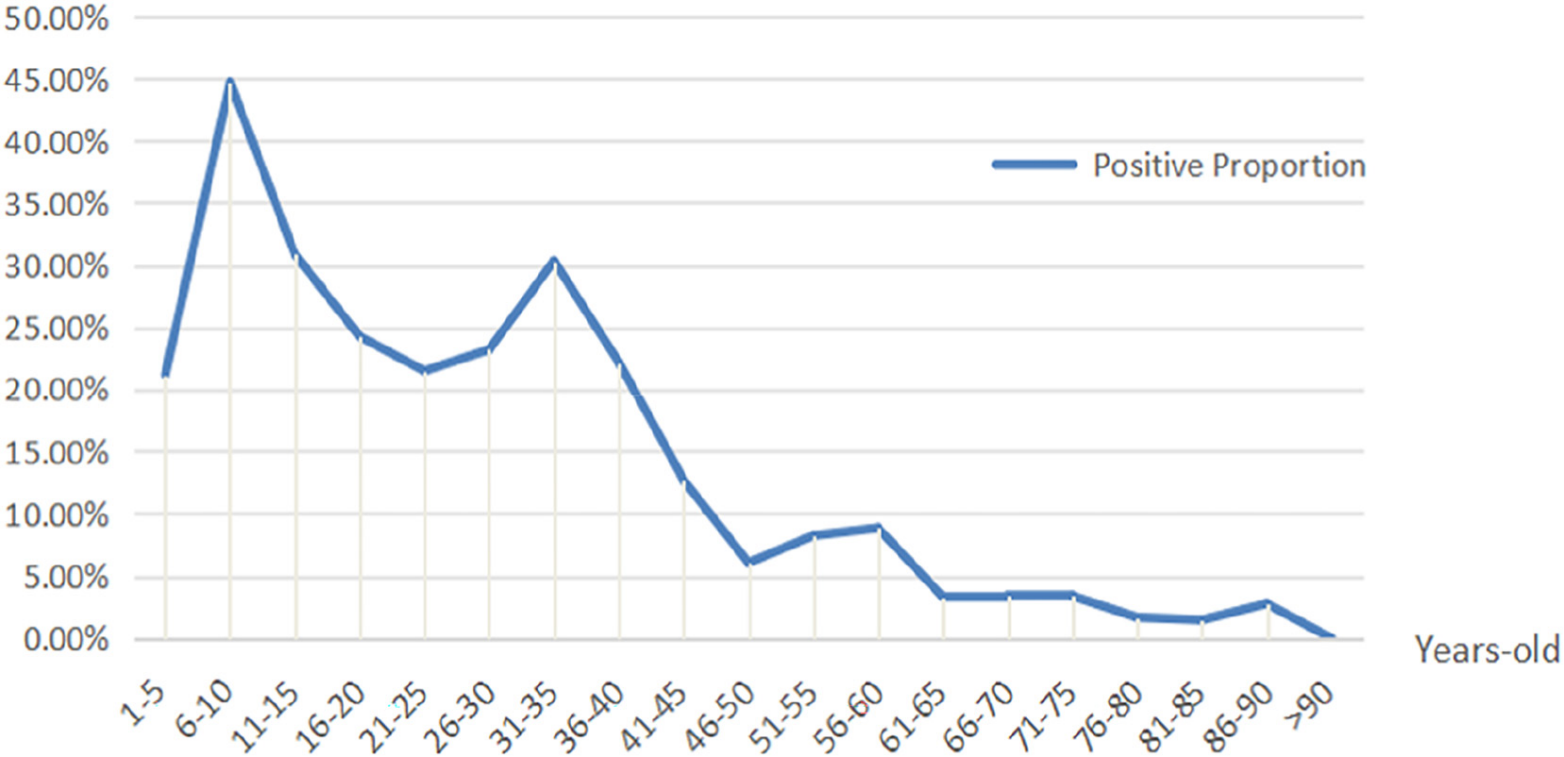
Positivity rates of *Mycoplasma pneumoniae* infection across different age groups in 7,234 patients presenting with respiratory tract infection symptoms. Data are shown as the percentage of PCR-positive cases within each age group (stratified in 5-year intervals from 1–5 years to 86–90 years, with a final group >90 years).

The NS samples were collected from 7,234 outpatients and inpatients (median age: 36 years) presenting with respiratory infection symptoms between June 2023 and June 2024, including 3,008 children (median age: 5 years) and 4,226 adults (median age: 60 years). Of these, 1,401 (19.37 %) cases were tested positive for *M. pneumoniae*, including 985 (32.7 %) children and 416 (9.84 %) adults, with a statistically significant difference observed between these two groups (p<0.001, Table 1). In addition, the female adults had a significantly higher infection rate than the male adults (12.3 4% vs. 7.34 %, p<0.001), while there was no marked difference in the infection rate between the sexes among pediatric patients (Table 1). The monthly distributions of *M. pneumoniae* infections are shown in Figure 1. A distinct epidemic peak was observed between July and December 2023, with monthly positivity rates ranging from approximately 21.49 % to 35.33 %. Subsequently, the infection rates showed minor fluctuations from April to June 2024, suggesting a transitional epidemiological phase. We also analyzed the distribution of *M. pneumoniae* infections across different age groups. As displayed in Figure 2, the highest infection rate for pediatric patients occurred in the 6–10 age group, while that for adults occurred in the 31–35 age group.

**Table 1:** Analysis of *Mycoplasma pneumoniae* infection in children and adults.

Groups	Positive percentage		p-Value
Children	32.75 %	(985/3,008)^a^	<0.001
Male	33.69 %	(561/1,665)^b^	0.2258
Female	31.57 %	(424/1,343)	
Adults	9.84 %	(416/4,226)	<0.001
Male	7.34 %	(155/2,111)^b^	
Female	12.34 %	(261/2,115)	

^a^Compare with the adult group, ^b^compare with the female group in children and adults.

### Difference in infectious indices between *M. pneumoniae* -positive and -negative patients in pediatric and adult populations

We investigated whether there was a difference in infectious indices between *M. pneumoniae* DNA-positive and -negative patients in pediatric and adult populations. As shown in Table 2, significant differences were observed in white blood cell (WBC) count, neutrophil count/proportion, lymphocyte count/proportion, CRP levels, PCT levels, and IL-6 levels between *M. pneumoniae*-positive and *M. pneumoniae*-negative pediatric patients. In contrast, in adults, only lymphocyte count and CRP levels exhibited statistically significant differences between *M. pneumoniae* DNA-positive and DNA-negative individuals.

**Table 2:** Differences in infectious indices between *Mycoplasma pneumoniae* (*MP*)-positive and -negative patients in pediatric and adult populations.

Variables	Reference range	Adults			Children		
		*MP*-negative	*MP*-positive	p-Value	*MP*-negative	*MP*-positive	p-Value
Age	–	63(43, 73)	36(30, 53)	<0.001	5(3, 7)	6(5, 8)	<0.001
WBC (10^9^/L)	3.69–9.16	6.06(4.78, 7.86)	5.95(4.94, 7.47)	0.681	6.7(5.42, 8.69)	7.41(5.83, 10.05)	0.036
Neutrophil count (10^9^/L)	2.04–7.5	4.07(2.95, 5.69)	4.06(3.05, 5.24)	0.467	3.01(2.02, 4.60)	3.95(2.96, 5.39)	<0.001
Neutrophil proportion (%)	50–70	68(59, 76)	68(59, 74)	0.080	44(33, 56)	54(47, 63)	<0.001
Lymphocyte count (10^9^/L)	0.8–4.0	1.30(0.93, 1.71)	1.34(1.04, 1.77)	0.032	3.11(2.21, 4.25)	2.59(1.75, 3.75)	<0.001
Lymphocyte proportion (%)	20–40	21(15, 30)	22(17, 29)	0.345	47(34, 57)	36(28, 44)	<0.001
CRP (mg/L)	0–5	10.00(10.00, 26.19)	27.80(10.05, 49.87)	<0.001	3.00(3.00, 8.40)	3.00(3.00, 4.00)	<0.001
PCT (ng/mL)	0–0.08	0.06(0.04, 0.11)	0.07(0.05, 0.10)	0.995	0.15(0.09, 0.28)	0.12(0.08, 0.20)	<0.001
IL-6 (pg/mL)	0–7.0	8.33(3.99, 20.3)	10.38(4.59, 18.82)	0.217	6.52(2.97, 13.83)	9.60(5.36, 17.32)	<0.001

### Analysis of antibiotic resistance to four antibiotic classes

Subsequently, we randomly selected 484 samples from the positive cases for *M. pneumoniae* culture and then obtained 78 culture-positive isolates (44 from children and 34 from adults). Antimicrobial susceptibility testing was conducted to determine the susceptibility of these isolates to four major antibiotic classes (macrolides, tetracyclines, quinolones, and lincosamides). As shown in Table 3, approximately 20.51 % (16/78) of isolates remained susceptible to all tested antibiotics, while 24.36 % (19/78) demonstrated resistance to a single antibiotic class. Among these single-class resistant isolates, macrolide resistance predominated (15.38 %, 12/78), followed by quinolones (6.41 %, 5/78) and tetracyclines (2.56 %, 2/78). However, these phenotypic resistance results should be interpreted cautiously. Moreover, multidrug resistance was observed in 55.13 % (43/78) of isolates, most of which (34.62 %, 27/78) were resistant to macrolides and lincosamides (clindamycin). Additionally, 6.41 % (5/78) of isolates exhibited resistance to all four antibiotic classes tested. These findings highlight the substantial prevalence of antibiotic resistance, particularly multidrug resistance patterns, among circulating *M. pneumoniae* strains in our study population.

**Table 3:** Antimicrobial susceptibility testing.

Resistance type		Count (n=78)	Percentage, %
Multidrug resistance	Macrolides & clindamycin	27	34.62 %
	Macrolides & tetracyclines	2	2.56 %
	Macrolides & quinolones	3	3.85 %
	Macrolides & clindamycin	1	1.28 %
	Tetracyclines & quinolones	2	2.56 %
	Macrolides & clindamycin & quinolones	1	1.28 %
	Macrolides & clindamycin & tetracyclines	2	2.56 %
	Macrolides & clindamycin & tetracyclines & quinolones	5	6.41 %
Resistance to a single antibiotic class	Macrolides	12	15.38 %
	Tetracyclines	2	2.56 %
	Quinolones	5	6.41 %
	Clindamycin	0	0.00 %
Non-resistant		16	20.51 %

### Antimicrobial susceptibility profiles of *M. pneumoniae* isolates

**Figure 3:**
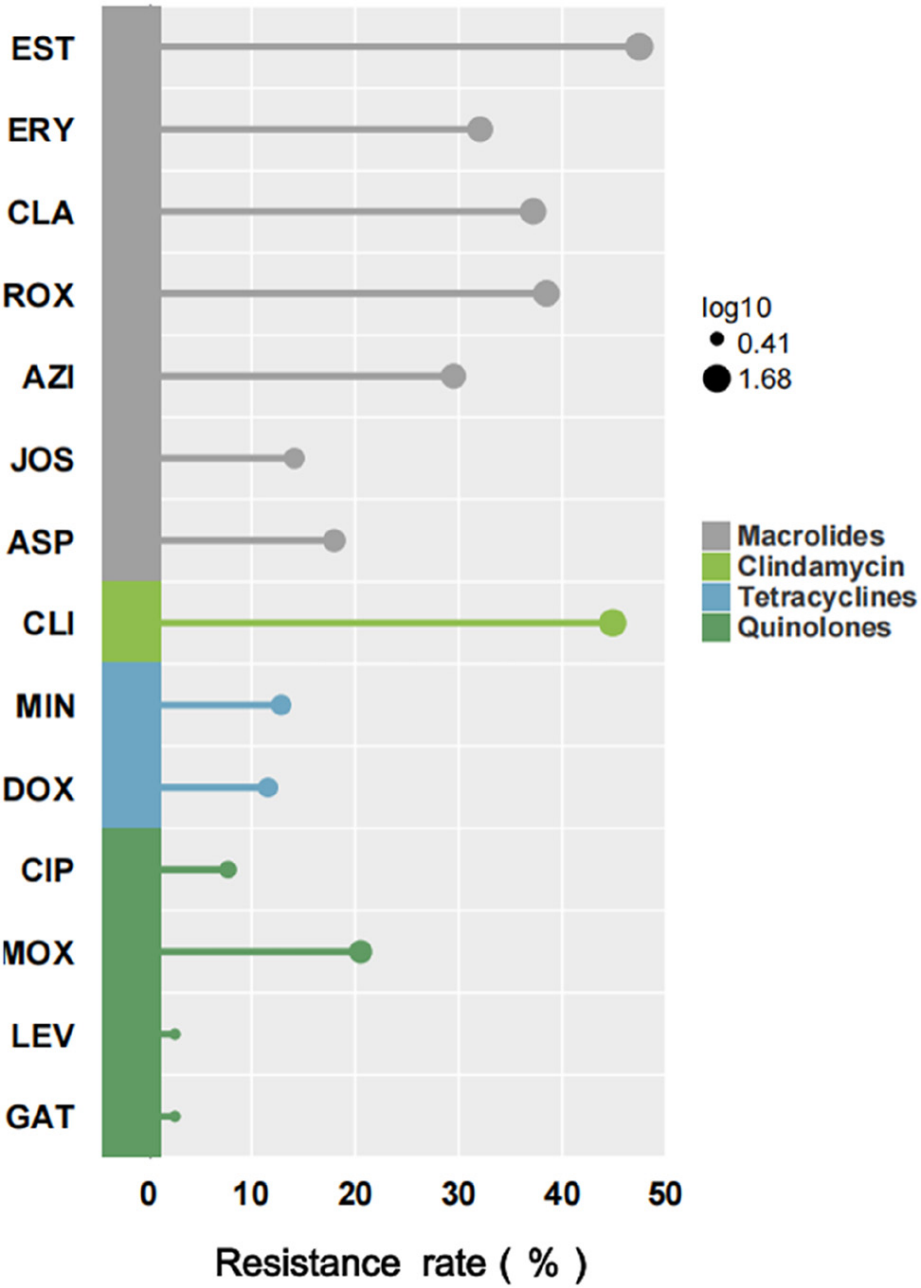
Phenotypic resistance rates to 14 antimicrobial agents in 78 culture-positive *Mycoplasma pneumoniae* isolates. The lollipop chart displays the percentage of resistant isolates for each agent, with antibiotics grouped into four classes: macrolides (erythromycin estolate [EST], erythromycin [ERY], clarithromycin [CLA], roxithromycin [ROX], azithromycin [AZI], josamycin [JOS], acetylspiramycin [ASP]), lincosamides (clindamycin, CLI), tetracyclines (minocycline [MIN], doxycycline [DOX]), and quinolones (ciprofloxacin [CIP], moxifloxacin [MOX], levofloxacin [LEV], gatifloxacin [GAT]). Resistance was interpreted according to the manufacturer’s instructions.

The susceptibility of 78 *M. pneumoniae* isolates to 14 antimicrobial agents across the four antibiotic classes was tested. As summarized in Figure 3 and Table 4, *M. pneumoniae* isolates had varying degrees of resistance to 14 antimicrobial agents. The highest resistance was observed for erythromycin estolate (EST) (47.44 %), followed by clindamycin (44.87 %). Notably, resistance to 14-membered ring macrolides was more prevalent than to tetracyclines and quinolones, while 16-membered ring macrolides exhibited lower resistance rates than their 14- and 15-membered counterparts. Among tetracyclines, minocycline (MIN) and doxycycline (DOX) demonstrated comparable resistance rates. In the quinolone class, moxifloxacin (MOX) displayed the highest resistance (20.51 %), in contrast to ciprofloxacin (CIP, 8 %), levofloxacin (LEV, 2.56 %), and gatifloxacin (GAT, 2.56 %). Additionally, 81.25 % (13/16) of MOX-resistant isolates originated from adult patients, whereas only 18.75 % (3/16) were from pediatric cases. This discrepancy may be attributed to the frequent use of MOX in the adult respiratory and critical care settings at our institution, whereas its use in pediatrics is restricted to cases where macrolide therapy has proven ineffective. Furthermore, intermediate resistance was most common in tetracyclines (44.23 %), followed by 16-membered ring macrolides (22.44 %). This phenomenon warrants heightened attention, as it underscores the need for clinicians to strictly adhere to antibiotic prescribing guidelines to curb the further development of resistance.

**Table 4:** Resistance and intermediate rates of 14 antimicrobial agents.

Antibiotic classes		Antimicrobial agents	Resistance (n)	Intermediate (n)	Sensitivity (n)	Resistance rate, %	Intermediate rate, %	Average resistance rate, %	Average intermediate rate, %
Macrolides (14/15/16-membered ring)	14	EST	37	0	41	47.44 %	0.00 %	38.78 %	3.85 %
		ERY	25	5	48	32.05 %	6.41 %		
		CLA	29	3	46	37.18 %	3.85 %		
		ROX	30	4	44	38.46 %	5.13 %		
	15	AZI	23	8	47	29.49 %	10.26 %	29.49 %	10.26 %
	16	JOS	11	23	44	14.10 %	29.49 %	16.03 %	22.44 %
		ASP	14	12	52	17.95 %	15.38 %		
Lincosamides		CLI	35	11	32	44.87 %	14.10 %	44.87 %	14.10 %
Tetracyclines		MIN	10	34	34	12.82 %	43.59 %	12.18 %	44.23 %
		DOX	9	35	34	11.54 %	44.87 %		
Quinolones		CIP	6	17	55	7.69 %	21.79 %	8.33 %	16.03 %
		MOX	16	9	53	20.51 %	11.54 %		
		LEV	2	9	67	2.56 %	11.54 %		
		GAT	2	15	61	2.56 %	19.23 %		

EST, erythromycin estolate; ERY, erythromycin; CLA, clarithromycin; ROX, roxithromycin; AZI, azithromycin; JOS, josamycin; ASP, acetylspiramycin; CLI, clindamycin; MIN, minocycline; DOX, doxycycline; CIP, ciprofloxacin; MOX, moxifloxacin; LEV, levofloxacin; GAT, gatifloxacin.

### Mutation analysis of 23S rRNA gene loci in cultured *M. pneumoniae* isolates

We performed targeted sequencing of four key loci (A2063G, A2064G, A2067G, and C2617G) in the 23S rRNA gene of 78 culture-positive isolates. As shown in Table 5, the overall mutation rate was 94.87 % (74/78). Among single-point mutations, A2063G was predominant, detected in 65.38 % (51/78) of isolates, while A2064G and C2617G single mutations were rare, each occurring in only 2.56 % (2/78) of cases. Among combined mutation patterns, the A2063G + C2617G double mutation was the most frequent (11.54 %, 9/78), followed by the A2063G + A2064G (7.69 %, 6/78). Notably, we identified only one isolate with a triple mutation involving (A2063G + A2064G + A2067G), and no isolates exhibited a single A2067G mutation. Comparative analysis revealed no significant differences in mutation type distribution between children and adults (p>0.05).

**Table 5:** Result of 23S rRNA mutation site type in the 78 cultured *Mycoplasma pneumoniae* isolates.

23S rRNA mutation	Groups (n=78)	Mutation (n, %)	p-Value
A2063G	Total	51(65.38 %)	0.912
	Children(n=44)	29(65.91 %)	
	Adults(n=34)	22(64.71 %)	
A2064G	Total	2(2.56 %)	0.187
	Children(n=44)	0(0.00 %)	
	Adults(n=34)	2(5.88 %)	
C2617G	Total	2(2.56 %)	0.502
	Children(n=44)	2(4.55 %)	
	Adults(n=34)	0(0.00 %)	
A2063G + A2064G	Total	6(7.69 %)	0.921
	Children(n=44)	4(9.09 %)	
	Adults(n=34)	2(5.88 %)	
A2063G + A2067G	Total	3(3.85 %)	0.819
	Children(n=44)	1(2.27 %)	
	Adults(n=34)	2(5.88 %)	
A2063G + C2617G	Total	9(11.54 %)	0.083
	Children(n=44)	8(18.18 %)	
	Adults(n=34)	1(2.94 %)	
A2063G + A2064G + A2067G	Total	1(1.28 %)	0.436
	Children(n=44)	0(0.00 %)	
	Adults(n=34)	1(2.94 %)	
No mutation	Total	4(5.13 %)	0.069
	Children(n=44)	0(0.00 %)	
	Adults(n=34)	4(11.76 %)	

## Discussion

In the present study, we found that children were more susceptible to *M. pneumoniae* infections than adults. Moreover, our study revealed a high prevalence of MRMP in adults, with the A2063G mutation being the dominant resistance mechanism.

During the COVID-19 pandemic, *M. pneumoniae* infection rates have decreased due to a wide range of non-pharmaceutical interventions. Nevertheless, a resurgence of *M. pneumoniae* cases has been observed in China following the relaxation of containment measures in December 2022 [12]. Unlike previous studies focusing solely on pediatric or adult populations, this study analyzed both concurrently. Previous studies have demonstrated that the incidence of *M. pneumoniae* infections is particularly high in school-aged children and adolescents [13], 14]. Consistently, our study revealed that the infection rate among children (32.75 %) was significantly higher than that among adults (9.84 %). In addition, this infection rate exceeded the previously reported rate of 20.7 % in Hubei Province during the COVID-19 pandemic (2021–2022) [15], confirming that the relaxation of COVID-19 restrictive measures has led to the resurgence of *M. pneumoniae* infections. Moreover, we found that children aged 6–10 years had the highest positivity rate of all pediatric cases. This susceptibility may be attributed to their developing immune systems and high contact rates in congregate classroom environments [16]. No significant gender differences were observed in pediatric infection rates. However, among adults, females exhibited a markedly higher infection rate (12.34 %) than males (7.34 %), which may be related to their occupational exposure variations or hormonal influences on immune response, though this requires further investigation. Furthermore, we found that infection-related indicators also varied: in children, all indicators tested showed significant differences between *M. pneumoniae* DNA-positive and -negative cases, whereas in adults, only lymphocyte count and CRP levels differed significantly. This disparity indicates that pediatric infection triggers broader immune activation, potentially explaining their more severe clinical presentations [17].

Previous evidence has suggested the difference in epidemic seasons among regions in China. In Southern China, *M. pneumoniae* infections are more common in summer and autumn, because hot weather may allow *M. pneumoniae* to survive longer in the environment and spread further [18]. Consistent results were observed in the present study. Our monthly analysis revealed that infections rose from June 2023, peaked at 35.33 % in November 2023, and declined in December 2023, confirming a seasonal pattern of *M. pneumoniae* epidemic. These results underscore the need for precautions such as reducing crowding and wearing masks during periods of high *M. pneumoniae* infections, especially in summer and autumn.

Macrolides such as azithromycin and clarithromycin are generally considered first-line treatment for *M. pneumoniae* infections [8]. Nevertheless, the widespread use of macrolides has resulted in the increase of MRMP, which can progress to severe pneumonia and extrapulmonary complications [19]. Herein, we evaluated the susceptibility of 78 clinical *M. pneumoniae* isolates to four classes of antibiotics. The results revealed that only 16 isolates were susceptible to all four classes of antibiotics, yielding an overall resistance rate of 79.5 % (62/78). Among the four antibiotic classes, macrolides exhibited the highest resistance rate (67.95 %), followed by clindamycin (46.15 %). Notably, all clindamycin-resistant isolates were also resistant to macrolides. This co-resistance pattern is consistent with the known effect of 23S rRNA mutations on macrolide–lincosamide–streptogramin B (MLSB)-like resistance [20]. Resistant *M. pneumoniae* strains can diminish the efficacy of antimicrobial therapies, prolong fever duration, extend hospital stays, and exacerbate disease severity [21], highlighting the need for heightened clinical awareness and careful consideration of antibiotic prescribing practices.

Notably, phenotypic resistance to tetracyclines and quinolones was observed in a small proportion of isolates (2.56 % and 6.41 %, respectively). However, in the absence of minimum inhibitory concentration (MIC) values and molecular analysis of known resistance determinants (e.g., gyrA/parC for quinolones or tetM for tetracyclines), these findings require cautious interpretation. Up to now, clinical isolates of *M. pneumoniae* have consistently shown near-100 % susceptibility to these antibiotic classes in China [22]. The observed phenotypic resistance in our commercial kit may reflect assay sensitivity limitations or *in vitro* variations rather than clinically relevant resistance mechanisms. Future studies incorporating MIC determination and targeted gene sequencing are warranted to confirm these observations. In addition, we acknowledge that the 78 culture-positive isolates may not fully represent the entire PCR-positive cohort due to the inherently low culture positivity rate of *M. pneumoniae*, which is consistent with the fastidious nature of this pathogen. The proportion of pediatric cases was lower in cultured isolates (56.41 %, 44/78) compared to the full PCR-positive cohort (70.31 %, 985/1,401; Fisher’s exact test p=0.011), indicating a slight under-representation of pediatric cases. This may reflect differences in bacterial load, specimen quality, or organism viability between pediatric and adult samples. However, the cultured isolates still included substantial numbers from both age groups (44 children and 34 adults).

Notably, the antimicrobial resistance patterns of *M. pneumoniae* also differed between age groups. Resistance rates to macrolides and clindamycin were higher in children, and quinolone resistance predominated in adults. This discrepancy may be attributed to restricted quinolone use in pediatric patients under 18 years owing to cartilage development risks [23]. No significant difference was observed in tetracycline resistance between the two groups. Among macrolides, 16-membered ring macrolides exhibited lower resistance (16.03 %) compared with 14- and 15-membered ring macrolides (38.78 % and 29.49 %, respectively), consistent with previous evidence [19]. Consequently, 16-membered ring macrolides, such as midecamycin, are recommended as first-line treatments for *M. pneumoniae* infections [24]. Within quinolones, moxifloxacin resistance was notably higher in adults (38.23 %) than in children (6.8 %), which is likely due to its preferential use in adult respiratory care and limited pediatric application. These findings highlight that *M. pneumoniae* resistance patterns to different antibiotics may vary depending on regional or departmental prescribing practices.

Due to the prolonged culture time required for *M. pneumoniae*, routine *in vitro* antimicrobial susceptibility testing is impractical for all cases but is recommended for immunocompromised patients, treatment failures, or novel antimicrobial evaluations [25]. Regular antimicrobial susceptibility testing for *M. pneumoniae* isolates from different departments could inform local resistance patterns and guide targeted antibiotic selection. The rising macrolide resistance has increased reliance on tetracyclines and quinolones as second-line agents [8]. However, their intermediate resistance rates in this study exceeded those of 14- and 15-membered-ring macrolides, indicating an emerging concern. While Guo et al. reported no clinical isolates resistant to quinolones in their study [26], the sequencing of quinolone resistance-associated genes (gyrA and parC) revealed multiple mutations in nucleotide sequences, necessitating ongoing genotypic and phenotypic surveillance.

In this study, sequencing of 78 isolates revealed a mutation rate of 94.87 % in the 23S rRNA V domain, higher than previously reported rates in Hubei (82.12 % in 2021 and 87.94 % in 2022) [15]. We found that the A2063G mutation was most prevalent (65.38 %), consistent with established mechanisms of macrolide resistance in *M. pneumoniae*. In addition, dual and triple mutations were also detected, indicating the importance of continuous mutation monitoring to track emerging trends and inform antimicrobial resistance management strategies. Although a formal statistical correlation between specific mutation types (e.g., A2063G single or dual mutations) and phenotypic resistance profiles was not performed, the high overall mutation rate (94.87 %) aligned closely with the predominant macrolide and macrolide-lincosamide co-resistance observed in susceptibility testing.

It is worth noting that this study has several limitations. First, the relatively small number of cultured isolates (n=78) limits the precision of resistance estimates, particularly for less common patterns such as tetracycline and quinolone resistance. Larger multicenter studies with higher culture yields are needed to confirm whether the low-level non-macrolide resistance observed in this study represents true emergence or sporadic events. Second, the susceptibility results in this study were interpreted based on the manufacturer’s criteria, which is a common practice in similar studies. However, differences in commercial kits and interpretation criteria may limit direct comparison of resistance rates across studies. Third, only major mutation sites (A2063G, A2064G, A2067G, C2617G) were analyzed in this study, potentially underestimating the mutation rate. Fourth, although basic laboratory parameters (e.g., WBC, IL-6 levels) were available, detailed clinical symptom profiles, disease severity, treatment response, and outcomes (e.g., fever duration) were not systematically collected. This precludes any correlation between resistance patterns (phenotypic or genotypic) and clinical manifestations or treatment outcomes, thereby limiting the direct clinical applicability of our findings. Additionally, all samples were obtained from a single hospital, which may limit the generalizability of these findings to other regions or populations. Future studies are required to address these limitations.

## Conclusions

This study investigated an *M. pneumoniae* outbreak in Wuhan from June 2023 to June 2024, peaking between July and December 2023. Children aged 5–8 years and adults aged 31–35 years were the most affected. Moreover, our study revealed a high prevalence of macrolide resistance in *M. pneumoniae*-infected adults, with the A2063G mutation being the dominant resistance mechanism. Therefore, establishing a long-term and systematic surveillance program is crucial for monitoring *M. pneumoniae* resistance patterns and genetic mutations, as well as for advancing epidemiological research.
